# Efficacy of classification-based cognitive functional therapy in patients with non-specific chronic low back pain: A randomized controlled trial

**DOI:** 10.1002/j.1532-2149.2012.00252.x

**Published:** 2012-12-04

**Authors:** K Vibe Fersum, P O’Sullivan, JS Skouen, A Smith, A Kvåle

**Affiliations:** 1 Physiotherapy Research Group Department of Public Health and Primary Health Care, University of BergenNorway; 2 School of Physiotherapy, Curtin UniversityBentley, Western Australia, Australia; 3 The Outpatient Spine Clinic Department of Physical Medicine and Rehabilitation, Haukeland University HospitalBergen, Norway

## Abstract

**Background**: Non-specific chronic low back pain disorders have been proven resistant to change, and there is still a lack of clear evidence for one specific treatment intervention being superior to another.

**Methods**: This randomized controlled trial aimed to investigate the efficacy of a behavioural approach to management, classification-based cognitive functional therapy, compared with traditional manual therapy and exercise. Linear mixed models were used to estimate the group differences in treatment effects. Primary outcomes at 12-month follow-up were Oswestry Disability Index and pain intensity, measured with numeric rating scale. Inclusion criteria were as follows: age between 18 and 65 years, diagnosed with non-specific chronic low back pain for >3 months, localized pain from T12 to gluteal folds, provoked with postures, movement and activities. Oswestry Disability Index had to be >14% and pain intensity last 14 days >2/10. A total of 121 patients were randomized to either classification-based cognitive functional therapy group *n* = 62) or manual therapy and exercise group (*n* > = 59).

**Results**: The classification-based cognitive functional therapy group displayed significantly superior outcomes to the manual therapy and exercise group, both statistically (*p* < 0.001) and clinically. For Oswestry Disability Index, the classification-based cognitive functional therapy group improved by 13.7 points, and the manual therapy and exercise group by 5.5 points. For pain intensity, the classification-based cognitive functional therapy improved by 3.2 points, and the manual therapy and exercise group by 1.5 points.

**Conclusions**: The classification-based cognitive functional therapy produced superior outcomes for non-specific chronic low back pain compared with traditional manual therapy and exercise.

## 1. Introduction

The current evidence for management of non-specific chronic low back pain (NSCLBP) reveals that interventions such as manual therapy, exercise, acupuncture, spinal injections and cognitive behavioural therapy are not superior to each other and have a limited long-term impact on the disorder (Assendelft et al., 2004; Furlan et al., 2005; Hayden et al., 2005; Ostelo et al., 2005; Staal et al., 2008).

Possible reasons for the failure of current clinical practice to effectively manage NSCLBP are proposed to lie in two main domains:

1. The failure to adequately deal with NSCLBP within a multidimensional biopsychosocial framework (Borkan et al., 2002). It has been proposed that NSCLBP represents a vicious cycle associated with different combinations of provocative factors. These include cognitive factors (such as negative beliefs, fear-avoidance behaviours, catastrophizing, hypervigilance, anxiety, depression, stress, poor pacing and maladaptive coping) (Vlaeyen and Crombez, 1999; Linton, 2000), physical factors (pain provocative postures and movement patterns related to altered body schema, muscle guarding, pain behaviours and deconditioning) (O’Sullivan et al. 2006b) and lifestyle factors (sedentary behaviour, inactivity and sleep deficits) (Bjorck-van Dijken et al., 2008).
2. Lack of a multidimensional classification system (MDCS) directing person-centred targeted management for this large group of NSCLBP patients. The Cochrane Back Review Group proposed that identification of subgroups is a key priority of low back pain (LBP) management in order to deal with the problem of patient heterogeneity (Bouter et al., 2003). Recent research supports this claim, with evidence that NSCLBP subjects can be broadly classified based on psychological factors (Turk, 2005; Boersma and Linton, 2006; Hill et al., 2010), movement and postural behaviours (Dankaerts et al., 2009), neurophysiological factors (Smart et al., 2011) and lifestyle behaviours [sedentary (Bjorck-van Dijken et al., 2008) vs. excessive activity (Mitchell et al., 2010)]. In spite of this knowledge, a recent systematic review concluded that few clinical trials exist utilizing MDCS or targeted interventions for NSCLBP (Fersum et al., 2010). The few trials that have taken a targeted biopsychosocial approach to the management of NSCLBP have demonstrated a tendency for improved outcomes (Klaber Moffett et al., 2004; Vollenbroek-Hutten et al., 2004; Asenlof et al., 2005; Riipinen et al., 2005).

A novel MDCS for LBP has been developed incorporating the biopsychosocial model (O’Sullivan, 2005). This system is integrated within the Quebec classification system (Spitzer, 1987) and represents a multilevel patient-centred clinical reasoning approach to broadly classify and target management for patients with NSCLBP. This person-centred intervention is called classification-based ‘cognitive functional therapy’ (CB-CFT) as it directly challenges these behaviours in a cognitively integrated, functionally specific and graduated manner. This MDCS has good inter-tester reliability (Dankaerts et al., 2006; Fersum et al., 2009), with a number of studies supporting the validity of the different subgroups on the physical domains (O’Sullivan et al., 2006a,b; O’Sullivan and Beales, 2007a,b; Beales et al., 2009; Dankaerts et al., 2009), as well as cognitive domains (Boersma and Linton, 2006). This MDCS has not been formally tested in a randomized controlled trial (RCT) for NSCLBP disorders. Therefore, the aim of this study was to investigate the efficacy of CB-CFT compared with manual therapy and exercise (MT-EX) for the management of NSCLBP. The hypothesis was that a person-centred classification-based cognitive functional approach to management of NSCLBP disorders would be more effective than MT-EX approach.

## What’s already known about this topic?

- Effect sizes from randomized controlled trials utilizing conservative treatment for non-specific chronic low back pain are small.
- Stratified treatment seems to be warranted and has socio-economic impact.
- For non-specific chronic low back pain, there does not seem to be a clear consensus as to which classification system to use.

## What does this study add?

- First trial for non-specific chronic low back pain utilizing a novel multidimensional classification system and cognitive functional approach.
- Cognitive functional approach shows clinical and statistically significant large effect sizes for primary outcomes as well as for secondary outcomes across multiple dimensions.

## 2. Methods

### 2.1. Study design

The study was a RCT designed to evaluate the efficacy of the CB-CFT according to the system proposed by O’Sullivan (2005), as compared with MT-EX in patients with NSCLBP. The effects of the intervention were assessed at 3- and 12-month follow-up. The study received ethical approval by the regional ethics committee of medical research in Western Norway (REK Vest). The clinical trial’s register number is NCT01129817.

### 2.2.Participants and procedures

A Norwegian university town, Bergen, with 250,000 inhabitants, provided the setting for the study. The patients were recruited from March 2006 to June 2008 from private physiotherapy outpatient practices, general practitioners and the outpatient spine clinic at the Haukeland University Hospital. In addition, six advertisements were placed in the local newspaper. All patients were presented with written information about the study with its aims and procedures. Here, it was clearly stated that there were two active comparable treatment arms, and that based on current knowledge, we did not know which was superior. [Correction added on 26 December 2012, after first online publication: Sentence has been corrected to read ‘… based on current knowledge, we did not know which was superior’]. The patients gave a written informed consent prior to proceeding to the clinical examination. The participants were eligible for the study if they were between the ages of 18 and 65 years, were diagnosed with NSLBP for >3 months that was primarily localized from T12 to gluteal folds, and they reported that their pain was provoked and relieved with postures, movement and activities. Pain intensity measured with a pain intensity numerical rating scale (PINRS) over the last 14 days >2/10 and an Oswestry Disability Index (ODI) > 14% was necessary to be admitted to the study.

The inclusion criteria of localized back pain with mechanical behaviour to the pain were designed to include patients whose movement behaviours had a clear association with their pain disorder, for which the CB-CFT intervention was designed.

The exclusion criteria were continuous sick-leave duration for >4 months, as it was considered that specific work-related intervention would have been required, acute exacerbation of LBP at time of testing in order to avoid regression to the mean, specific LBP diagnosis (radicular pain, disc herniation, spondylolisthesis, stenosis, Modic changes), any low limb surgery in the last 3 months, surgery involving the lumbar spine, pregnancy, diagnosed psychiatric disorder, widespread constant non-specific pain disorder, pain without a clear mechanical behaviour, active rheumatologic disease, progressive neurological disease, serious cardiac or other internal medical condition, malignant diseases, acute traumas, infections or acute vascular catastrophes. A lack of compliance of greater than 50% was set as a withdrawal criterion based on programme compliance forms. An a priori power calculation suggested that 63 patients in each group would give 80% power to detect a group difference of 5 points in the ODI [estimating a standard deviation (SD) of 10 points in both groups], and 1 point on the PINRS at 1-year post-intervention (estimating an SD of 2 points in both group).

The randomization took place at the University of Bergen (UiB). A person independent of the study developed a randomization schedule and produced 160 sealed opaque envelopes containing each participant’s allocation. Randomization was performed in permuted blocks of 16. When the patients had been examined and classified and a blinded examiner had collected baseline data, the patient drew the envelope containing their allocation and details of procedure in relation to their allocation.

All subjects first underwent a comprehensive interview and full physical examination at the Department of Public Health and Primary Health Care, UiB. This multidimensional examination was important in order to broadly classify each subject based on his or her pain provocative postures and movement behaviours, lifestyle and cognitive behaviours (O’Sullivan, 2005). During the interview, subjects were guided in questioning to inform: their history of pain, pain area and nature, pain behaviour (aggravating/easing movements and activities), their primary functional impairments, disability levels, activity levels and sleep patterns. Inquiries were also made regarding their level of fear of pain and any avoidance of activities, work and social engagement. Their degree of pain focus, pain coping strategies, stress response and its relationship to pain, and their pain beliefs were also questioned as was any history of anxiety and depression. Finally, their beliefs and goals regarding management of their disorder were ascertained. This information was considered alongside the Orebro Musculoskeletal Pain Questionnaire (OMPQ) (Linton and Boersma, 2003) where the patient reported high levels (>5) of stress/anxiety, depressed mood, fear of movement and lack of pain coping strategies. These factors were then considered within the context of the movement behaviours and lifestyle factors, i.e., in the context of fear, avoidance of physical activity and specific pain provocative movement patterns were assessed.

The physical examination involved analysis of the subject’s primary functional impairments (pain provocative postures, movements and functional tasks reported during the interview), assessment of their body control and awareness, as well as easing postures and movements (O’Sullivan, 2005).

The physical examination process involved a systematic process of assessment of pain provocative postures (such as sleeping, sitting, standing and bending) and functional movement tasks (such as sit to stand, single-leg stand, spinal movements and lifting) and any other specific tasks nominated by the patient as pain provocative or that they avoided. The validity of this clinical examination approach has been demonstrated in a number of studies (O’Sullivan et al., 2006a,b; Dankaerts et al., 2007; O’Sullivan and Beales, 2007a,b; Beales et al., 2009; Dankaerts et al., 2009; Sheeran et al., 2012). The reliability has previously been reported for both physical and cognitive aspects based on the health-care practitioners’ ability to synthesize the patient story and clinical examination to broadly classify the patient, based on identification of the primary drivers of the disorder within the different levels of the MDCS (Dankaerts et al., 2006; Fersum et al., 2009). The examination process follows a systematic approach (as outlined in Supporting Information Appendix S1), but is tailored to the patients’ presentation. In Supporting Information Appendix S2, all the included subjects and their subsequent classification are outlined.

The intervention took place at 3 different private clinics and lasted for 12 weeks. Patients were followed-up immediately and 12 months post-intervention. After the 12-week intervention period, participants were permitted to seek alternative care, and the frequency and type of treatment were monitored in the follow-up questionnaires. A tester blinded to allocation, pretreatment and at 3-month follow-up distributed the questionnaires. During the 12-month follow-up, the questionnaires were sent by mail.

### 2.3. Interventions

#### 2.3.1. CB-CFT

The behavioural management approach provided was based on the MDCS described by O’Sullivan (2005) and Fersum et al. (2009). Three experienced physiotherapists who conducted the management had undergone, on average, 106 hours of CB-CFT training (including workshops, patient examinations, pilot study and clinical manual). Depending on the different levels of the classification system, each patient received a specific targeted intervention directed at changing their individual cognitive, movement and lifestyle behaviours considered by the therapist to be maladaptive (provocative) of their disorder. Physiotherapists were also guided by the individuals’ OMPQ in order to better target their management within the psychosocial domain. The CB-CFT intervention has four main components: (1) a cognitive component, for each patient, their vicious cycle of pain was outlined in a diagram based on their findings from the examination and the OMPQ; (2) specific movement exercises designed to normalize maladaptive movement behaviours as directed by the movement classification; (3) targeted functional integration of activities in their daily life, reported to be avoided or provocative by the patient; and (4) a physical activity programme tailored to the movement classification (see Supporting Information Appendix S3 for detailed description of the CB-CFT). The initial session was 1 h and follow-ups were 30–45 min. Patients were seen at a weekly basis for the first two to three sessions and then progressed to one session every 2–3 weeks during the 12-week intervention period.

All instructions for subjects were written, and they were asked to complete the programme on a daily basis and complete a daily diary outlining if they had complied with each aspect of the intervention.

#### 2.3.2. MT-EX

Participants allocated to the comparison group were treated with joint mobilization or manipulation techniques applied to the spine or pelvis consistent with best current manual therapy practice. These therapists were specialists in orthopaedic manual therapy with an average of 25.7 years of experience with no prior training in the use of the MDCS or CB-CFT. The particular dose and techniques were at the discretion of the treating therapist, based on each participant’s examination findings. In addition, most patients (82.5%) in this group were given exercises or a home exercise programme. This included general exercise or motor control exercise, but not based on the specific MDCS as outlined by O’Sullivan (2005). The motor control exercises involved isolated contractions of the deep abdominal muscles in different functional positions as previously described (Richardson et al., 1998). The therapists in this group generally spent 1 h with the patients for the initial consultation and 30 min for follow-ups.

### 2.4. Treatment fidelity

Several strategies were applied to enhance treatment fidelity. For every patient, a report was written, documenting type of treatment, number of treatments, medication usage before and after the intervention. In addition, there was a therapist session-by-session documentation of treatment content as well as individual working sheets. The mean number of treatments was 7.7 in the CB-CFT group (range 4–16; SD 2.6), and 8.0 in the MT-EX group (range 3–17; SD 2.9). All the treating physiotherapists in both treatment arms prior to the intervention underwent half a day of training with a clinical psychologist regarding the concepts of best-practice cognitive approach to managing back pain in order to standardize this for both intervention arms as per clinical guidelines. Therapy was in the primary care setting for both groups and was pragmatic. Treatment was discontinued if the therapist deemed the participant had no further need of treatment before the 12 weeks were completed, as is standard clinical practice. There was no difference between the two groups in terms of medication intake before or after the treatment.

### 2.5.Outcome measures

#### 2.5.1. ODI

The primary outcome measurement for perceived function was the ODI (Roland and Fairbank, 2000). The ODI psychometric properties have been well established (Fairbank and Pynsent, 2000). Studies have shown good construct validity (Greenough, 1993; Strong et al., 1994), acceptable internal consistency and test–retest reliability (Kopec et al., 1996), and high responsiveness (Deyo and Centor, 1986).

#### 2.5.2. Pain

Pain intensity in the previous week was another primary outcome, measured by the PINRS (Jensen et al., 1986). The PINRS measures pain severity by asking the patient to select a number (from 0 to 10) to represent how severe the pain had been over the last 2 weeks. PINRS is more reliable than the visual analogue scale, especially with less educated patients (Ferraz et al., 1990).

#### 2.5.3. Hopkins Symptoms Checklist

Secondary outcome measures for anxiety and depression were Hopkins Symptoms Checklist (HSCL-25) (Derogatis et al., 1974). It consists of 25 items: part I of the HSCL-25 has 10 items for anxiety symptoms; part II has 15 items for depression symptoms. Optimal HSCL-25 cut-off was 1.67 for men and 1.75 for women (Sandanger et al., 1998).

#### 2.5.4.Fear-Avoidance Beliefs Questionnaire (FABQ)

The fear-avoidance beliefs of physical activity and work were measured using the FABQ (Waddell et al., 1993). The reliability and validity of this measure has been reported previously (Kovacs et al., 2006).

#### 2.5.5. Total lumbar spine range of motion

Total lumbar spine range of motion was measured with the two inclinometer method (Mayer et al., 1984) utilizing standardized protocol (Adams et al., 1986; Bullock-Saxton, 1993; Chaory et al., 2004). The true lumbar motion was obtained from a subtraction of the pelvic motion from the gross motion expressed in angular degrees of flexion and extension (Adams et al., 1986).

#### 2.5.6. Patient satisfaction

The patients filled out a patient satisfaction questionnaire (Ware and Davies, 1983) at 3- and 12-month follow-up. This was just a simple questionnaire from 1 to 5 asking the patients how satisfied they were with their treatment: 1 = satisfied, 2 = just a little satisfied, 3 = neither satisfied nor dissatisfied, 4 = just a little dissatisfied, 5 = dissatisfied.

#### 2.5.7.Sick-leave days

In case of sick-leave days, this was extracted from the Ørebro Screening Questionnaire using a 10-category variable. The question asked: ‘How many days of work have you missed because of pain during the last 18 months?’ The 10 categories were merged into 3. First category was 0 days, second category was 1–7 days and third category was >7 days.

#### 2.5.8. Care-seeking

Questions regarding subsequent treatments between the 3-month intervention period and long-term follow-up were also assessed with a questionnaire at 12 months. The patients were asked the following questions: (1) Have you had any treatment since the intervention finished? Yes/No; (2) What type of treatment have you had?; and (3) How many treatments have you had?

### 2.6. Statistical considerations and analysis

A linear mixed model was used to estimate the group differences in treatment effect at both time points and also in the change in outcome from 3- and 12-month follow-up, with baseline values included as the only covariate. Age, gender, body mass index (BMI), LBP duration and work status were evaluated as possible confounders, but did not need to be included in the final models. Bootstrapped standard errors were estimated to adjust for departures from normality, as some outcome measures displayed slightly skewed distributions. Models were examined to confirm the absence of influential outlying observations. Statistical significance was set at *p* = 0.05.

Analysis was performed on an ‘available case’ basis. Two subjects (one CB-CFT and one MT-EX) were missing data at 3-month follow-up but provided 12-month data, and five subjects (two CB-CFT and three MT-EX) were missing 12-month follow-up data but provided 3-month data. These cases were included in the model, as the linear mixed model used is a likelihood-based estimation procedure resulting in non-biased estimates, provided data are missing at random. Statistically significant group differences in sick-leave days, patient satisfaction and number of treatments sought post-intervention were assessed using two-sample Wilcoxon rank sum (Mann–Whitney) test. In the case of sick-leave days, the original variable from Ørebro Screening Questionnaire collapsed down for tabular display but analysed using original 10-category variable, i.e., 0, 1–2, 3–7, …, etc., days.

In addition, change in pre- to post-treatment was estimated in both treatment groups using paired *t*-tests, and the change in ODI and PINRS were calculated for each study participant, in terms of absolute change from baseline, and tabulated with reference to consensus values for minimally important change (MIC) in these outcomes.

All authors had full access to the data in the study and the corresponding author had final responsibility to submit for publication. An independent person, unfamiliar with the aim, groups and content of the study, entered data into a database. Statistical analysis was performed using Stata/IC 10.1 for Windows (StataCorp LP, College Station, TX, USA). A secondary independent statistician blinded to the treatment group allocation and unfamiliar with the aim, groups and content of the study also reviewed the statistical analysis.

## 3. Results

**Figure 1:**
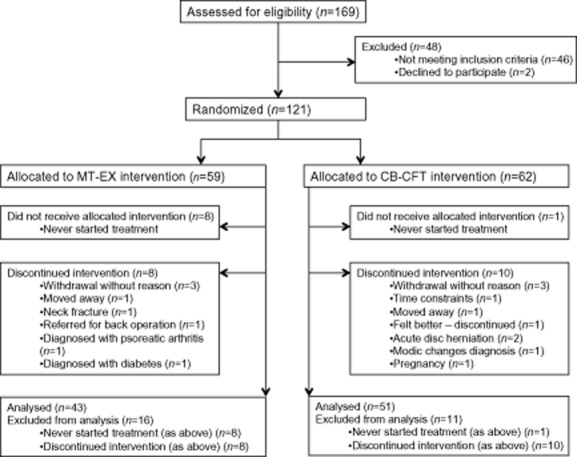
Flow chart depicting participant recruitment and finalenrolment for the two groups: manual therapy and exercise (MT-EX) and classification-based cognitive functionaltherapy (CB-CFT).

Out of the 169 patients initially enrolled, 121 patients met the inclusion criteria and were found eligible. In the randomized cohort,62 patients were assigned to the CB-CFT group, and 59 were assigned to the MT-EX group (Fig. 1). A total of 16 of 59 (27.1%) patientsassigned to MT-EX and 11 of 62 (17.7%) patients assigned to CB-CFT either did not start treatment or did not complete treatment,and were unavailable for either 3- or 12-month follow-up assessment, which precluded an intention-to-treatanalysis.Table 1 displays the baseline characteristics of these patients, by treatment allocation.

**Table 1:** Baseline characteristics of study participants

	Analysed (*n* = 94)		Excluded from analysis (*n* = 27) (did not start or discontinued treatment)	
	MT-EX (*n* = 43)	CB-CFT (*n* = 51)	MT-EX (*n* = 16)	CB-CFT (*n* = 11)
Characteristics				
Age – mean (SD)	42.9 (12.5)	41.0 (10.3)	39.9 (13.8)	37.0 (10.5)
Female – *n* (%)	21 (48.8)	27 (52.9)	10 (62.5)	5 (45.5)
LBP duration – *n* (%)^a^				
3–12 months	6 (14.3)	6 (11.8)	0	0
1–5 years	13 (31.0)	14 (27.5)	4 (30.8)	1 (12.5)
>5 years	23 (54.8)	31 (60.8)	9 (69.2)	7 (87.5)
Height (cm) (SD)	173.5 (8.6)	174.3 (9.4)	171.8 (10.1)	177.4 (7.9)
Weight (SD)	76.2 (13.1)	78.3 (16.6)	76.9 (16.5)	85.4 (11.9)
BMI (SD)	25.2 (3.5)	25.6 (4.0)	25.9 (4.3)	27.1 (3.1)
Work status				
Paid work *n* (%)	34 (79.1)	45 (88.2)	15 (93.8)	11 (100.0)
Primary outcomes – mean (SD)				
Oswestry Disability Index	24.0 (8.0)	21.3 (7.5)	27.9 (7.6)	23.1 (8.1)
Pain intensity last week	5.3 (1.9)	4.9 (2.0)	6.2 (1.2)	6.0 (1.9)
Secondary outcomes – mean (SD)				
Hopkins Symptoms Checklist	1.57 (0.39)	1.40 (0.33)	1.78 (0.53)	1.60 (0.42)
Fear-avoidance physical	11.8 (5.0)	11.1 (3.9)	12.6 (4.6)	10.9 (5.3)
Fear-avoidance work	19.3 (11.1)	14.1 (9.6)	17.8 (8.2)	13.5 8.7
Sick-leave days – *n* (%)				
None	13 (30.2)	15 (29.4)	2 (12.5)	3 (27.3)
1–7 days	9 (20.9)	13 (25.5)	4 (25.0)	2 (18.2)
>7 days	21 (48.9)	23 (45.1)	10 (62.5)	6 (54.5)
Lumbar ROM – mean (SD)	46.2 (13.0)	50.2 (14.9)	44.3 (13.6)	57.6 (9.8)

Disability: Oswestry Disability Index (0–100), low scores indicate low disability.

Pain: intensity last week (pain intensity numerical rating scale 0–10), low scores indicate low pain intensity.

Anxiety/depression: Hopkins SymptomsChecklist – 25-item checklist – low scores indicate low anxiety and depression.

Fear of movement/(re) injury: The Fear-Avoidance Beliefs – Physical (0–24), low scores indicate low fear related to physical activities.

Fear of movement/(re) injury: The Fear-Avoidance Beliefs –Work (0–42), low scores indicate low fear related to work activities.

Range of motion: Lumbar motion was obtained from a subtraction of the pelvic motion from the gross motion expressed in angular degrees of flexion and extension.

BMI, body mass index; CB-CFT, classification-based cognitive functional therapy; LBP, low back pain; MT-EX, manual therapy and exercise; ROM, range of motion; SD, standard deviation.

a Missing four cases from MT-EX and three from CB-CFT group.

Analysed study participants in the two treatment arms were comparable in terms of baseline characteristics, with the exception of small but significant differences in HSCL and FABQ work (Table 1).

Both groups significantly improved with the respective therapeutic interventions. After adjustment for baseline scores, the CB-CFT group displayed superior outcomes supported by both statistically and clinically significant differences when compared with the MT-EX group. This was evident both immediately after and at 12-month post-intervention for both primary and secondary outcomes(Table 2). This was demonstrated by the degree of improvement in the CB-CFT group for ODI score being 13.7 points [95% confidence interval (CI): 11.4–16.1; *p* < 0.001] and for PINRS scores 3.2 (95% CI: 2.5–3.9; *p* < 0.001). In the MT-EX group, the mean improvement for ODI score was 5.5 points (95% CI: 2.8–8.3; *p* < 0.001) and 1.5 for PINRS (95% CI: 0.7–2.2; *p* < 0.001). The improvements for all secondary outcomes showed similar effects, with the CB-CFT group demonstrating significantly greater change when compared with the MT-EX group across all outcomes, except for total lumbar range of motion (Table 2).

**Table 2:** Outcomes (unadjusted means and SDs)

	MT-EX		CB-CFT		CB-CFT vs MT-EX^a^
	Mean	SD	Mean	SD	Mean difference (95 % CI)
Primary outcome variables					
Oswestry Disability Index Questionnaire					
Baseline	24.0	8.0	21.3	7.5	
3 months	18.5	8.1	7.6	6.7	−9.7 (−12.7 to −6.7)****
12 months	19.7	11.7	9.9	9.8	−8.2 (−12.6 to −3.8)****
Pain intensity in last week					
Baseline	5.3	1.9	4.9	2.0	
3 months	3.8	1.9	1.7	1.7	−2.1 (−2.7 to −1.4)****
12 months	3.8	2.1	2.3	2.0	−1.3 (−2.1 to −0.5)****
Secondary outcome variables					
Hopkins Symptoms Checklist					
Baseline	1.56	0.39	1.40	0.33	
3 months	1.43	0.37	1.20	0.27	−0.12 (−0.19 to −0.04)***
12 months	1.51	0.47	1.22	0.32	−0.13 (−0.22 to −0.04)***
Fear-avoidance physical					
Baseline	11.8	5.0	11.1	3.9	
3 months	10.3	6.0	6.1	5.0	−3.6 (−5.3 to −1.9)****
12 months	10.9	5.5	5.8	5.5	−4.7 (−6.5 to −3.0)****
Fear-avoidance work					
Baseline	19.1	11.1	14.1	9.6	
3 months	17.4	10.8	8.3	8.4	−5.7 (−7.8 to −3.6)****
12 months	16.6	12.2	7.7	9.0	−5.6 (−8.7 to −2.5)****
Total lumbar spine range of motion					
Baseline (degrees)	46.2	13.0	50.2	14.9	
3 months	45.6	12.7	49.7	14.0	1.9 (−2.8 to 6.7)

	MT-EX			CB-CFT			
Sick-leave days [*n* (%)]	0	1–7	>7	0	1–7	>7	
Baseline	14 (31.8)	9 (20.5)	21 (47.7)	15 (29.4)	13 (25.5)	23 (45.1)	
12 months	16 (40.0)	7 (17.5)	17 (42.5)	32 (65.3)	7 (14.3)	10 (20.4)	*z* = 2.95**

Patient satisfaction^b^ [*n* (%)]	MT-EX						CB-CFT											
	1	2	3	4	5	1	2	3	4	5
3 months^3^	28 (68.3)	6 (14.6)	5 (12.2)	0	2 (5.9)	48 (94.1)	1 (2.0)	2 (3.9)	0	0	*z* = 3.21**
12 months^3^	18 (46.2)	8 (20.5)	9 (23.1)	3 (7.7)	1 (2.5)	46 (95.8)	1 (2.1)	1 (2.1)	0	0	*z* = 5.18***

Care-seeking after intervention [*n* (SD) ]	MT-EX	CB-CFT	
12 months	10.6 (13.3)	2.1 (5.4)	*z* = 4.79****

Disability: Oswestry Disability Index (0–100), low scores indicate low disability.

Pain: intensity last week (pain intensity numerical rating scale 0–10), low scores indicate low pain intensity.

Anxiety/depression: Hopkins Symptoms Checklist – 25-item checklist – low scores indicate low anxiety and depression.

Fear of movement/(re) injury: The Fear-Avoidance Beliefs – Physical (0–24), low scores indicate low fear related to physical activities.

Fear of movement/(re) injury: The Fear-Avoidance Beliefs – Work (0–42), low scores indicate low fear related to work activities.

Range of motion: Lumbar motion was obtained from a subtraction of the pelvic motion from the gross motion expressed in angular degrees of flexion and extension.

CB-CFT, classification-based cognitive functional therapy; MT-EX, manual therapy and exercise; SD, standard deviation.

a Negative values favour CB-CFT.

b Patient satisfaction: (1–5), 1 = completely satisfied, 2 = a little bit satisfied, 3 = neither satisfied or dissatisfied, 4 = a little bit dissatisfied, 5 = completely dissatisfied.

** *p* < 0.01.

*** *p* < 0.001.

Table 3 presents the improvements in primary outcome measures in terms of absolute improvement from baseline. There was maintenance of treatment effect over the 3- to 12-month follow-up time for both groups, with no significant main effect for time or group/time interaction identified in the linear mixed models.

**Table 3:** Immediate and long-term improvements in primary outcome measures from baseline [*n* (%)]

	3 months				12 months			
	MT-EX		CB-CFT		MT-EX		CB-CFT	
	*n*	%	*n*	%	*n*	%	*n*	%
Oswestry								
No change	14	33.3	2	4.0	12	30.8	5	10.2
<10 points	14	33.3	12	24.0	15	38.5	13	26.5
10–19 points	12	28.6	23	46.0	9	23.1	24	49.0
20–29 points	1	2.4	11	22.0	2	5.1	5	10.2
30 or more points	1	2.4	2	4.0	1	2.5	2	4.1
Pain (PINRS)								
≤0	15	35.7	6	12.0	12	30.8	10	20.4
1 point	8	19.1	6	12.0	11	28.2	3	6.1
2 points	7	16.7	9	18.0	7	17.9	14	28.6
3 points	4	9.5	8	16.0	4	10.3	7	14.3
4–5 points	4	9.5	13	26.0	3	7.7	7	14.3
>6 points	4	9.5	8	16.0	2	5.1	8	16.3

Minimally important change: Clinically meaningful changes are defined as >10-point change in Oswestry Disability Index and >1.5 on PINRS (Ostelo et al., 2008). CB-CFT, classification-based cognitive functional therapy; MT-EX, manual therapy and exercise; PINRS, pain intensity numerical rating scale.

## 4.Discussion

This study reveals that the CB-CFT group demonstrated superior outcomes compared with the MT-EX group across every domain measured at post-intervention and at 12-month follow-up (Table 2). Both groups showed significant improvement in short- and long-term follow-ups; however, the CB-CFT group was superior based on clinically meaningful changes as defined by MIC, defined as >10-point change in ODI and >1.5 on PINRS (Ostelo et al., 2008).

The effect sizes of conservative interventions from previous Cochrane reviews reveal findings similar to the MT-EX group (Assendelft et al., 2004). In response, calls have been made for a paradigm shift, away from a biomedical ‘injury’ model, to viewing LBP as a multifactorial biopsychosocial disorder, and directing treatment at beliefs and behaviours that promote pain and disability rather than simply at the signs and symptoms associated with the disorder. Calls have also been made for the need for a multidimensional classification-based approach to direct management of NSCLBP in order to make treatment more person-centred (Borkan et al., 2002; O’Sullivan, 2011). This is supported by reports that disability levels in chronic pain are better predicted by cognitive and behavioural aspects of pain rather than sensory and biomedical ones (Campell and Edwards, 2009). CB-CFT addresses all of these objectives.

Although satisfaction rates were high in both groups, odds of being completely satisfied were over three times higher in the CB-CFT group at 3 months and five times higher at 12 months. The degree of patient satisfaction is seen as a reflection of the quality of care and as an important outcome in its own right (May, 2001). The importance of communication, patient-centred approaches and goal matching has been well documented in the literature as important for the therapeutic relationship, enhancing compliance and patient outcomes (Linton, 2005; O’Sullivan, 2011).

CB-CFT had a strong cognitive focus with an emphasis on reframing the persons’ understanding of their back pain in a person-centred manner, with an emphasis on changing maladaptive movement, cognitive and lifestyle behaviours contributing to their vicious cycle of pain. This was performed by means of reflective communication, providing a contemporary understanding of pain mechanisms, correcting faulty pathoanatomical beliefs, goal setting, verbal, written and visual feedback (viewing their own back) and a strong emphasis on normalizing movement behaviours within a graded functional approach.

Although this is the first RCT to address maladaptive movement behaviours specific to the patient’s presentation within a cognitive framework, the exact benefits from targeting specific movement training cannot be isolated from the other aspects of the intervention. The behaviours that were targeted were prioritized based on the movements or postures that patients reported that they most feared, avoided and/or that provoked them. These identified movements were the targets for the movement retraining aspect of the intervention based on the patient’s classification and were integrated to the goals of the patient. The use of visual feedback such as mirrors was central to this process. The aim of this approach was for each subject to acquire self-management strategies for their disorder by developing positive back pain beliefs, pain control, developing adaptive strategies of movement that enhanced functional capacity and the ability to engage in regular physical activity.

These findings are supported by previous reports of benefits with different targeted behavioural approaches to managing NSCLBP. Moseley et al. (2004) reported reduced pain and enhanced function associated with pain education. Asenlof et al. (2009) reported superior long-term outcomes for treating NSCLBP with an individually tailored behavioural intervention targeting cognitions, motor behaviour and activity, compared with physical therapy. The use of visual feedback when training movement in patients with LBP has also been shown to reduce pain and influence functional capacity (Sheeran et al., 2012; Wand et al., 2012). Hill et al. (2011) reported superior outcomes when management was targeted on the basis of psychosocial risk factors. Vlaeyen et al. (2002) reported benefits with a graded exposure approach to management in a series of NSCLBP patients with high levels of fear. CB-CFT incorporates all of these aspects within its intervention.

Given the multidimensional nature of the CB-CFT intervention, it is not clear as to the exact basis for the superior outcomes. We hypothesize that the mechanisms for change are likely to be multifactorial given the patient-centred body–mind behavioural approach, in contrast to a more treatment-orientated signs and symptoms approach in manual therapy. On one hand, this behavioural approach may have impacted on cognitive factors known to affect pain sensitivity and disability such as developing positive beliefs, reduced fear, increased awareness, enhanced understanding and control of pain, adaptive coping, enhanced self-efficacy, confidence and improved mood. Evidence for this is supported by the reduction in fear of movement and improved mood observed following the intervention. On the other hand, the functional behavioural aspects of the intervention were targeted at enhancing body awareness, relaxation of guarded muscles, normalizing maladaptive movement patterns, body schema retraining with the use of mirror feedback, extinguishing pain behaviours, conditioning and increased functional capacity. These factors have been associated with levels of pain, disability, fear and catastrophizing (Wideman et al., 2009; Lewis et al., 2012; Wand et al., 2012). We also acknowledge that the active engagement required of subjects for this behavioural approach may present a barrier for those unwilling to self-manage their disorder (Carr et al., 2006). This may be dependent on the levels of acceptance and readiness to engage in behavioural change, although these factors were not formally assessed.

Although it was not a primary aim of the CB-CFT intervention, the results demonstrate a 2.95-times less likelihood of taking sick leave for their LBP at 12 months compared with the MT-EX group. Previously, only studies using cognitive behavioural therapy in multidisciplinary treatment models have shown an effect on sick-listing for this patient group (Airaksinen et al., 2006). However, as these numbers were extracted from the OMPQ and were self-reported, these findings must be interpreted with some caution. The patients in the CB-CFT group also sought less additional treatment for their pain, implying they may have been more empowered to self-manage their disorder, suggesting significant cost–benefits.

A limitation of this study was the number of patients that either did not start or complete treatment. While there was a comparable proportion of non-completers in each group (Table 2), 8 of 59 (13.5%) of the MT-EX group failed to commence their allocated treatment, compared with only 1 of 62 (1.6%) of the CB-CFT group. This may be due to the fact that seven of these subjects had reported previous manual therapy treatment with poor effect, which would have potentially biased for a poorer outcome in the MT-EX group. It should be noted that there was no statistically significant difference between completers and non-completer based on baseline characteristics, and as we performed our analysis conditioning on baseline scores and confirmed the absence of a confounding effect of age, gender, BMI, LBP duration and work status on our results, we are confident that our estimates of treatment effect are not substantially biased by these missing cases. Also of note, no dropouts reported adverse effects from either intervention arm. Furthermore, due to a lack of power, we were unable to determine the influence of the subject classification on the outcome.

We acknowledge that there are also a number of additional methodological considerations that may have influenced the results of this study. Firstly, the patients were recruited from a variety of sources both primary and secondary care levels, as well as newspaper advertising, which could have influenced the kind of patients who entered the study. However, the wide inclusion criteria in the study suggest that a common and representative group of patients with chronic localized LBP without objective sign of pathology to the spine were included. Webb et al. (2003) reported that an Oswestry score of >25% is considered the cut-off score for classifying ‘disabling back pain’. The patients recruited were, on average, just below this [24.0 (MT-EX) and 21.3 (CB-CFT)] at baseline, and hence it can be said that the patients sampled in this study had moderate back pain and functional impairment sufficient to result in activity limitation and sick leave for many (see Table 1). It is also acknowledged that therapists in both arms of the study were not blinded to the intervention, and although all therapists had considerable experience, the influence of therapist enthusiasm and expectation for change was not controlled for.

Furthermore, the multidimensional nature of the study limits any conclusion as to the specific effects of the different components of the intervention. Future research that investigates matching versus non-matching of interventions for patients with chronic mechanical LBP may help identify the effects of specific aspects of the intervention (Kent et al., 2010). Also given this was a pragmatic trial, the intervention dose was not controlled in either group, although both groups received remarkably similar attention. While this intervention appears to be successful for the population we tested, further studies are needed to investigate those with higher levels of pain and disability, patients that are long-term sick-listed as well as in other cultural and occupational groups, in order to determine the generalizability of the findings. This approach also needs to be compared with other cognitive approaches and tested within a multi-centre trial framework in the future.

### 4.1. Conclusions

The results of this study support that a behaviourally orientated targeted approach to manage NSCLBP (CB-CFT) was more effective at reducing pain, disability, fear beliefs, mood and sick leave at long-term follow-up than MT-EX.

## Supporting Information

Appendix S1 Displays an overview of the multidimensional classification system. This system is guided by a clinical reasoning process that considers the presence and dominance of different factors, allowing for patient-centred targeted intervention.

Appendix S2 Displays an overview of the randomized subjects (*n* = 121) and how they spread out in the different categories.

Appendix S3 Schematic overview of the four different components of the CB-CFT treatment.
