# Supplementary material for: Efficacy of classification-based cognitive functional therapy in patients with non-specific chronic low back pain: A randomized controlled trial
Source: Eur J Pain. 2012 Dec 4;17(6):916–28. doi: 10.1002/j.1532-2149.2012.00252.x (PMC3796866; doi:10.1002/j.1532-2149.2012.00252.x)
Supplement: Appendix S1 — Displays an overview of the multidimensional classification system. This system is guided by a clinical reasoning process that considers the presence and dominance of different factors, allowing for patient-centred targeted intervention. [file ejp0017-0916-sd1.doc]

**Stage of disorder: acute (1-4wk), sub-acute (4-12 wk), chronic > 12 wk, recurrent episodic**

**Red flag disorders**

Cancer

Inflammatory disorders

Infections

Fractures

**Specific LBP**

Lateral canal stenosis

Central stenosis

Disc prolapse

Modic changes

Spondylolisthesis

*Radiology must correlate with clinical presentation*

**Non-specific LBP**

No clear patho-anatomical diagnosis

(Localized or generalized)

*Based on clinical examination and review of available radiology*

**Multi-dimensional classification system for axial low back pain**

(Pain dominant between T12 and buttocks)

**Pain with mechanical behaviour**

Pain that has a clear and consistent anatomical focus

Pain has a proportionate mechanical behavior

(Provoked and relieved with specific activities and postures)

**Pain with non-mechanical behaviour**

Spontaneous, constant generalized pain with no clear anatomical focus, either unrelated to mechanical factors or a disproportionate exaggerated and sustained pain response to minor mechanical triggers.

**Lifestyle and individual factors**

Patient questioned regarding:

1. Physical activity levels and preferences: frequency, duration, intensity levels per week as rated by patient, sedentary behaviours

2. Work related factors – heavy repeated loading, monotony, sedentary behaviours, ergonomic considerations

3. Sleep related problems and sleep hygiene, energy and vitality, smoking

4. Lifestyle - life stress events, current stress levels, the basis of it and their perceived levels of control over this

5. Goal setting regarding lifestyle factors, activities they enjoy, perceived barriers to activity, realistic physical activity levels to attain

**Cognitive and Psycho-social factors**

Patient questioned regarding:

1. *Beliefs* - regarding the basis of their pain, strategies they perceive will help them, advice they have been given, future consequences

2. *Fears* – regarding movement, activities, avoidance behaviours, work, future consequences of pain disorder

3. *Stress and anxiety* – current levels, enquire regarding its basis (social factors, underlying trait anxiety, pain related anxiety), stress responsiveness and how it impacts on pain and muscle tension, how this relates to their thoughts, fears and behaviours, levels of vigilance and focus on pain

4. *Mood* – levels of mood and how this impacts on their pain and lifestyle factors

5. *Coping strategies* – current coping strategies (avoidant vs. endurance), whether they have active coping strategies for their pain, their perceived ability to cope with and manage their pain, levels of pacing, willingness to engage in self management strategies

6. *Work / home* related factors – coping, levels of support, life stress, absenteeism, presenteeism, seeking compensation

7. *Co-morbidities* – psychological and general health history and perception

This aspect of the examination was integrated with the Orebro Screening Questionnaire (where score is >5 for related specific items these items were specifically explored with the patient)

**Pain related movement behaviours**

Identify and examine the primary pain provocative functional impairments (postures, movements and activities as reported by patient)

Identify the postural and/or movement control strategy adopted with the activity (posture and movement analysis)

Identify pain behaviours – avoidance (limb loading, activity or movement), propping with hands, breath holding, bracing etc

Identify levels of body relaxation, breathing control, body control, awareness and movement plasticity during provocative movements and postures

Determine whether the adopted strategy is provocative (mal-adaptive) or protective (adaptive) of the disorder

i.e. Does reinforcing the ‘patients’ strategy provoke their pain?

Determine how these behaviors relate to the patients sense of self (body schema), beliefs, fears, anxiety levels, coping strategies

Explore whether an alternative movement or postural control strategy be found via relaxation and postural adjustment, that reduces pain and / or facilitates functional capacity

Levels of conditioning, muscle strength and endurance specific to functional impairments

**Control impairment behaviour**

Pain associated with movement and/or loading in a specific movement direction or postural task.

***No movement impairment*** in direction of pain provocation during provocative activity and / or postural task.

Adapting the movement / control strategy to reduce focal spinal loading (postural adjustment and /or relaxation) during the provocative task *reduces the pain and enhances functional capacity*

Directions: Extension, flexion, multi-directional sensitivity

(Note: if predominantly a loading problem there may not be a direction of provocation)

**Movement impairment behaviour**

Pain associated with movement and/or loading in a specific movement direction or postural task.

***Movement impairment*** (guarding) in direction of pain provocation during provocative activity.

Graduated movement exposure into the direction of pain provocation with controlled motor relaxation *reduces pain* *and enhances functional capacity* during provocative task

Directions: Extension, flexion, multi-directional sensitivity

**Mixed presentation**
