# Supplementary figures and images for: Efficacy of classification-based cognitive functional therapy in patients with non-specific chronic low back pain: A randomized controlled trial
Source: Eur J Pain. 2012 Dec 4;17(6):916–28. doi: 10.1002/j.1532-2149.2012.00252.x (PMC3796866; doi:10.1002/j.1532-2149.2012.00252.x)

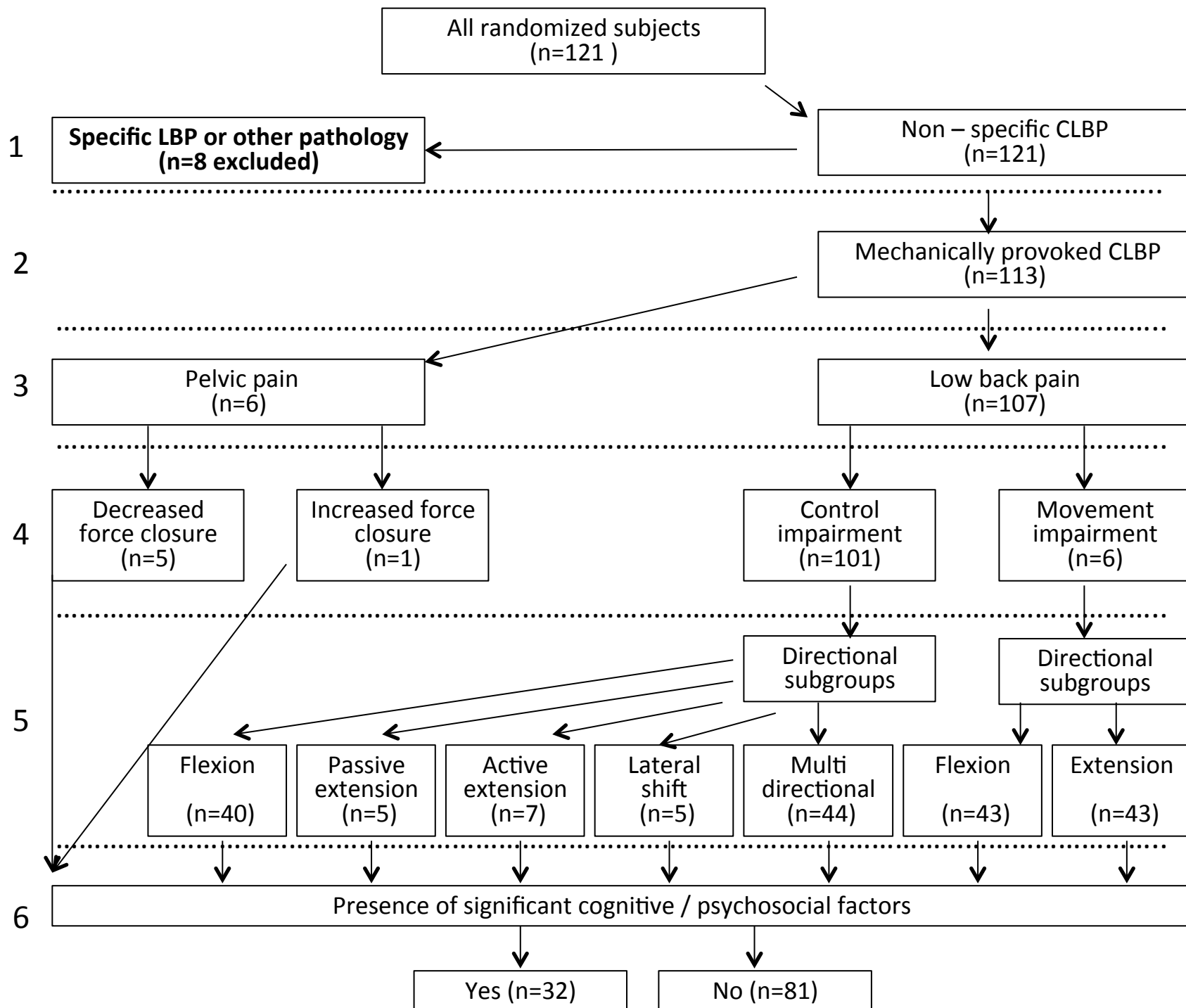

Supplement: Appendix S2 — Displays an overview of the randomized subjects (n = 121) and how they spread out in the different categories. [file ejp0017-0916-sd2.pdf]
