# Supplementary material for: Efficacy of classification-based cognitive functional therapy in patients with non-specific chronic low back pain: A randomized controlled trial
Source: Eur J Pain. 2012 Dec 4;17(6):916–28. doi: 10.1002/j.1532-2149.2012.00252.x (PMC3796866; doi:10.1002/j.1532-2149.2012.00252.x)
Supplement: Appendix S3 — Schematic overview of the four different components of the CB-CFT treatment. [file ejp0017-0916-sd3.docx]

| **Component** | **Description**  **All patients were asked to fill in a compliance questionnaire regarding the four aspects of the intervention and present it each session.** |
| --- | --- |
| 1.  Cognitive component | For each patient their vicious cycle of pain was outlined in a diagram based on their findings from the examination and the Orebro MPQ. For patients identified with more contributing psychosocial factors, greater emphasis was placed on this aspect of the intervention.  For example, it was explained how negative beliefs about pain, fear of movement, increased focus on pain, low mood and poor pacing reinforced avoidance and protective behaviours, muscle guarding, altered movement patterns and body postures, which in turn fed a vicious cycle of pain sensitisation and disability.  This process was openly discussed and the patient was invited to consider how they might be able to break their cycle and set their goals for management. These functional goals formed the basis on which the individual exercise management was developed and targeted in the context of lifestyle factors relevant to the individual.  Patients were also informed regarding neuro-physiological concepts of pain sensitisation, and that pain does not equal harm or structural damage. If patients had concerns regarding their radiological imaging, they were informed that radiological findings are common in pain free subjects, and correlate poorly with levels of pain and disability.  This aspect of the intervention was revisited throughout the treatment periods and integrated into the functional aspects of management as the patient was challenged and exposed to previously provocative tasks. For example with fear of movement, the patients beliefs about pain, perceived consequences and their behavioural responses to both the thoughts and act of the movement were examined. This allowed the patient to be mindful of fearful thoughts driving stress responses and protective behaviours during specific postures, movements and tasks. This reflection allowed them to modify these responses. All other aspects of the intervention listed below had a strong cognitive focus with an emphasis on reflective communication, self-management practices, functional enhancement and goal orientation.  Prior to discharge acute exacerbation management planning was discussed with each patient in order to promote an active / confrontation approach to pain management. |
| 2.  Functional movement exercises | The aim of this aspect of the intervention was to provide patients with alternative strategies to normalise their postural and movement behaviours.  If a patient was unable to relax their trunk muscles, they were initially instructed to learn diaphragm breathing in relaxed postures such as lying, sitting and standing as a first step before movement training.  Once this was achieved, all patients received targeted functional postural and movement training based on their movement classification and directed by the activities and postures that they either avoided due to pain or that provoked their pain.  This approach followed a ‘graded exposure’ model where the patient was exposed to previously pain provocative tasks, but in a relaxed and controlled manner, as dictated by their movement classification and with feedback (visual with the use of mirrors, mental imagery and awareness of body responses such as breath holding, muscle guarding and changes respiration rate).  For example, for those with a **‘movement impairment’** classification, patients were directed to move into the direction of pain provocation in a graded manner (first using less threatening movements and progressing to more threatening movements and activities) in order to restore normal unguarded movement. In the case of flexion ‘movement impairment’ disorder, this first involved gentle non-weight bearing lumbar flexion exercises progressing to flexion in sitting, standing and lifting, whilst ensuring that the movements were performed in a relaxed manner, without breath holding and protective behaviours.  For those with a **‘control impairment’** classification, subjects were trained to modify their pain provocative postures and movement patterns to reduce pain whilst performing the task. For example, for a flexion ‘control impairment’ provoked with sitting, bending and lifting, patients were taught to change their pattern of movement to reduce lumbo-sacral flexion during these tasks. They were first taught to dissociate lumbo-pelvic from thoracic movement in sitting to reduce lumbo-pelvic flexion. This was then progressed to bending and lifting with a focus on facilitating hip/pelvic and thoracic flexion in a relaxed manner ([O'Sullivan, 2005a](#_ENREF_58)).  The management of the few subjects with pelvic girdle pain, followed a similar approach, documented previously ([O'Sullivan and Beales, 2007a](#_ENREF_62), [b](#_ENREF_63)).  Simple non-threatening low load exercises were gradually progressed towards higher load and more complex functional exercises, as the subject gained confidence and control in performing the tasks. No more than 3 or 4 exercises were given at a time.  This challenged each patient to perform functional activities and postures that they nominated as pain and/or fear provoking without adopting pain behaviours such as grimacing, breath holding, muscle guarding, propping with hands or avoidance such as asymmetrical loading.  In this manner they were instructed to change old pain provocative movement behaviours and to reinforce their new functionally enhancing movement behaviours. This was augmented by dynamic practical demonstration of the postures or movement by the therapist, the use of mirrors so they could view their own spines to enhance body schema awareness, written instruction and stick body diagrams (outlining the ‘old way’ vs. the ‘new way’ of sitting, standing, bending, lifting and moving).  Pain related movement behaviours such as propping on the hands, breath holding and abdominal bracing were discouraged.  Where the patient’s functional goals required it, this was progressed into a conditioning program to build strength and endurance within these functional tasks. |
| 3.  Functional Integration | The exercise program in stage 2 was functionally integrated, specific to each persons nominated pain provocative activities and directed at their functional goals.  The aim was to restore normal functional movement capacity, enhanced body awareness, reduce avoidance, pain behaviours and fear by means of pain control and confrontation in daily life.  Here each participant nominated activities of daily living that provoked their pain and these were rehearsed with the therapist, so that they were confident and mindful of normalising their movement behaviours whilst performing these tasks in activities of daily living. If the participant reported activities they avoided, these were also rehearsed and they were encouraged to confront these activities (without protective behaviours) and include them in their daily activities. |
| 4. Physical activity levels | Patients were asked to carry out some form of physical exercise (such as walking or biking) 3-5 times a week if they weren’t previously doing so (the duration for this was initially based on the patients exercise tolerance and gradually increased to 20-40 minutes duration). They were encouraged to be mindful of how they performed this to facilitate normal movement in a relaxed manner. The type of the physical activity was directed both by the movement classification and the patient’s preference. For example, if the patient had an extension ‘control impairment’ disorder they were first asked to use an exercise bike or bicycle to until they had acquired sufficient control, conditioning and confidence to begin a graduated walking program. |
